# Acthar gel in the treatment of nephrotic syndrome: a multicenter retrospective case series

**DOI:** 10.1186/s12882-016-0241-7

**Published:** 2016-03-31

**Authors:** Arvind Madan, Snezana Mijovic-Das, Ana Stankovic, Geoffrey Teehan, Amber S. Milward, Anupa Khastgir

**Affiliations:** Nephrology Associates of Central Florida, 3885 Oakwater Circle, Orlando, FL 32806 USA; Albany Medical College, Albany, NY USA; HCA Inc. Physician Services, Salem, NH USA; Lankenau Medical Center, Wynnewood, PA USA; Nephrology Practice, 3366 NW Expressway, Bldg D, Suite 280, Oklahoma City, OK 73112 USA

**Keywords:** ACTH, Acthar gel, Nephrotic syndrome, Proteinuria

## Abstract

**Background:**

Current first-line anti-proteinuric treatments for nephrotic syndrome (NS) do not produce an effective response in all patients and are not tolerated by some patients. Additional effective and tolerable treatment options in NS are strongly needed. This retrospective case series is the largest to date to examine Acthar gel (adrenocorticotropic hormone, ACTH) in patients with varied-etiology NS.

**Methods:**

This multicenter retrospective case series included adult patients with NS (*N* = 44) treated with Acthar gel at 6 clinical practices. NS etiologies included idiopathic focal segmental glomerulosclerosis (FSGS, 15), idiopathic membranous nephropathy (iMN, 11), IgA nephropathy (IgAN, 5), diabetic nephropathy (DN, 4), systemic lupus erythematosus class V membranous lupus nephritis (MLN, 2), minimal change disease (MCD, 2), membranoproliferative glomerulonephritis (MPGN, 1), fibrillary glomerulonephritis (FGN, 1), and unbiopsied NS (3). Proteinuria response was assessed as percent reduction from baseline and percent of patients meeting complete remission (final proteinuria <500 mg/d), partial remission (≥50 % reduction in proteinuria from baseline and final proteinuria 500–3500 mg/d), clinical response (≥30 % reduction in proteinuria from baseline that did not meet criteria for complete or partial remission), and no response (failed to meet remission or clinical response criteria) following Acthar gel therapy. Safety and tolerability were examined using adverse event (AE) frequency reported by patients or treating nephrologists and frequency of early discontinuation of treatment due to AEs.

**Results:**

68.2 % (30/44) of patients had received prior NS treatment with immunosuppressive or cytotoxic therapies. Thirty-seven patients completed Acthar gel treatment. Seven patients (15.9 %) had early termination due to AEs, including weight gain (2), hypertension (2), edema (1), fatigue (1), seizures (1) and for reasons not stated (2). Proteinuria reduction ≥30 % was shown in 81.1 % (30/37) of patients and 62.2 % (23/37) showed ≥50 % proteinuria reduction. Proteinuria responses were greatest in MCD (*n* = 2/2 complete remission), MLN (*n* = 2/2 partial remission), MPGN (*n* = 1/1 partial remission), FSGS (*n* = 12/15 [80.0 %] partial remission or clinical response), and iMN (*n* = 8/11 [72.7 %] complete remission, partial remission, or clinical response).

**Conclusions:**

Acthar gel may meet an important treatment need in patients with treatment-resistant NS in response to first-line therapies, patients unable to tolerate first-line therapies, and in patients with advanced disease.

## Background

Patients with nephrotic syndrome (NS) show a combination of clinical and laboratory features of renal diseases characterized by heavy proteinuria, hypoalbuminemia, and peripheral edema, with hyperlipidemia also frequently seen. Effective treatment in patients who experience treatment resistance or relapse following initial immunosuppressive treatment with steroids or cytotoxic drugs is an ongoing challenge across NS etiologies [1–8]. There remains a strong need for effective, tolerable treatments for patients with treatment-resistant NS, particularly without the renal and extra-renal toxicities associated with many first- and second-line therapies [9]. One such treatment is H.P. Acthar® Gel (adrenocorticotropic hormone, repository corticotropin injection, Mallinckrodt ARD Inc., Hazelwood, MO), FDA-approved in the US to induce diuresis or remission of proteinuria in NS without uremia of the idiopathic type or that due to lupus erythematosus [10].

Adrenocorticotropic hormone (ACTH) treatment for NS emerged in the US in the 1950s and was shown to be effective for reduction or remission of proteinuria, depending on the dose and duration of ACTH treatment [11, 12]. However, by the late 1960s ACTH had largely been replaced by steroids in NS treatment due to the convenience of oral dosing and the belief that they had similar mechanisms of action and treatment effects [13]. Acthar gel treatment for NS, the only FDA-approved ACTH treatment in the US, has recently re-emerged [14–20].

Though current clinical data are limited, proteinuria reduction resulting in complete or partial remission following Acthar gel treatment has been shown in patients with idiopathic membranous nephropathy (iMN), idiopathic focal segmental glomerulosclerosis (FSGS), IgA nephropathy (IgAN), minimal change disease (MCD), and diabetic nephropathy (DN) [14–20]. Treatment regimens of 80 U Acthar gel twice weekly for 6 months were commonly used. One exception involved patients with iMN who received either a 40 U or 80 U dose twice weekly for either 3 months or 6 months, showing that greater reduction in proteinuria was associated with greater cumulative Acthar gel dose [19]. Additionally, patients with advanced DN were treated with Acthar gel 16 U or 32 U daily for 6 months [17].

Our retrospective case series is the largest published to date to examine the efficacy and safety of Acthar gel treatment in patients with varied-etiology NS who have typically failed multiple previous therapies. Proteinuria reduction as well as frequency of adverse events (AEs) in patients treated with Acthar gel within clinical practices were evaluated.

## Methods

### Patients

Patients eligible for inclusion were diagnosed with NS, ≥18 years old, received Acthar gel treatment for ≥6 months, and had assessment of either 24-h proteinuria level (mg/d) or urine protein:creatinine ratio (UPCR; g/g converted to mg/d) prior to and following 6 months of Acthar gel treatment. Patients did not have to meet a pre-specified level of proteinuria at baseline to be included in the study. NS etiologies within the patient cohort included idiopathic NS due to FSGS, MN, IgAN, MCD, membranoproliferative glomerulonephritis (MPGN), fibrillary glomerulonephritis (FGN), and 3 unbiopsied NS patients. Additionally, patients with systemic lupus erythematosus (SLE) class V membranous lupus nephritis (MLN), and patients receiving off-label treatment for NS due to DN were included. With the exception of the 3 unbiopsied patients, all patients had biopsy-confirmed NS etiology. The patient cohort represents all patients treated with Acthar gel from the participating clinical practices who met the specified inclusion criteria.

### Ethics

This multicenter, retrospective case series of prescription-based treatment with Acthar gel for NS included 6 US clinical practices. The study received institutional review board exemption from the New England Institutional Review Board. All data reported in this paper have be de-identified in order to protect patient confidentiality.

### Data reviewed

Clinical records were reviewed for demographic and clinical characteristics, including NS etiology, prior immunosuppressive or cytotoxic treatments, and levels of proteinuria, serum albumin, and total cholesterol. Hypoalbuminemia was defined as <3.5 g/dL. Renal function was evaluated using serum creatinine (SCr) level and renal insufficiency was defined as SCr >1.3 mg/dL. Acthar gel treatment dosing regimen and concomitant medications, including angiotensin II receptor blockers (ARBs), angiotensin-converting-enzyme inhibitors (ACEIs), and immunosuppressive and cytotoxic drugs, were documented.

Post-treatment proteinuria level and percentage reduction in proteinuria from baseline were examined. Consistent with prior studies, complete remission was defined as final proteinuria <500 mg/d, and partial remission was defined as ≥50 % reduction in proteinuria from baseline and final proteinuria 500–3500 mg/d, with examination of preserved or improved renal function as indicated by SCr that does not worsen >25 % from baseline [14–16]. Clinical response was defined as ≥30 % reduction in proteinuria from baseline that did not meet criteria for complete or partial remission. Inclusion of the clinical response outcome aligns with the treatment suggestion for calcineurin inhibitors (CNIs) in patients with iMN, stating therapy should be continued in patients showing an initial substantial proteinuria reduction of 30–50 % at 4–6 months treatment because the optimal treatment duration with calcineurin inhibitors is unknown and the patient’s response suggests possible further proteinuria improvement with continued therapy [2]. The clinical response definition is also consistent with the clinical practice of the current study’s treating nephrologists when determining the duration of a trial of treatment in patients. That is, treatment is extended following a ≥30 % reduction in proteinuria because this degree of improvement has been experienced as a clinically meaningful change for patients. Patients showing no response failed to meet remission or clinical response criteria.

The frequency of AEs and the frequency of early discontinuation of treatment due to AEs were documented. Patients with an early termination of treatment without a stated reason were included in the count of early termination due to AEs.

### Data analysis

Categorical variables were summarized using counts and percentages. Descriptive statistics summarized continuous variables. Paired t-tests examined change from baseline to post-Acthar gel therapy in proteinuria level, serum albumin, and total cholesterol. Between-group t-tests were used to compare percent reduction in proteinuria in patients who showed renal insufficiency at baseline versus patients who did not show renal insufficiency. Statistical analyses were performed using SAS (version 9.1; SAS Institute, Cary, NC).

## Results

### Study participants

Characteristics of patients by NS etiology group are presented in Table 1. Cases included 44 patients across NS etiologies FSGS (*n* = 15), iMN (*n* = 11), IgAN (*n* = 5), DN (*n* = 4), MLN (*n* = 2), MCD (*n* = 2), MPGN (*n* = 1), FGN, (*n* = 1), and 3 unbiopsied NS patients. The majority (30/44; 68.2 %) had failed ≥1 prior immunosuppressive or cytotoxic therapy, and 20 of 44 (45.5 %) had failed ≥2 prior immunosuppressive and/or cytotoxic treatments. All patients received Acthar gel 80 U twice weekly, with the exception of 1 patient with iMN who received 40 U twice weekly. Continuing treatment with standard care ARB and/or ACEI maximum blockade was received by 36/44 (81.8 %) patients (Tables 2, 3, 4 and 5; 12/15 FSGS, 9/11 iMN, 5/5 IgAN, 4/4 DN, 2/2 MLN, 2/2 MCD, 1/1 MPGN, 0/1 FGN, 1/3 unbiopsied NS) and dosing was maintained throughout the Acthar gel treatment period unless the patient required dose modification due to AEs.

**Table 1:** Demographic and clinical characteristics of NS patients (*N* = 44) treated with Acthar gel

NS etiology	Age ± SD, years	Gender, *n* (%) female	Race/ethnicity, *n* (%) White	Previous IST/CT, *n* (%) yes
FSGS (*n* = 15)	53.3 ± 12.9	7 (47)	12 (80)	12 (80)
iMN (*n* = 11)	53.6 ± 18.9	4 (36)	10 (91)	10 (91)
IgAN (*n* = 5)	35.0 ± 8.4	2 (40)	4 (80)	1 (20)
DN (*n* = 4)	54.0 ± 19.9	2 (50)	4 (100)	0
MLN (*n* = 2)	37.5 ± 4.9	1 (50)	0	2 (100)
MCD (*n* = 2)	33.5 ± 13.4	2 (100)	2 (100)	2 (100)
FGN (*n* = 1)	63.0	0	1 (100)	1 (100)
MPGN (*n* = 1)	22.0	1 (100)	1 (100)	0
Other^a^ (*n* = 3)	55.7 ± 6.1	2 (67)	2 (67)	2 (67)

*Abbreviations*: *CT* cytotoxic therapy, *DN* diabetic nephropathy, *FGN* fibrillary glomerulonephritis, *FSGS* idiopathic focal segmental glomerulosclerosis, *IgAN* IgA nephropathy, *iMN* idiopathic membranous nephropathy, *IST* immunosuppressive therapy, *MCD* minimal change disease, *MLN* membranous lupus nephritis (class V), *MPGN* membranoproliferative glomerulonephritis, *NS* nephrotic syndrome

^a^“Other” includes 3 patients with unbiopsied NS

**Table 2:** Proteinuria reduction and treatment response in patients with FSGS treated with Acthar gel

Patient	Previous IST/CT	Concurrent medications	Serum albumin	SCr	Proteinuria	Treatment response
				Pre-Acthar	Pre-Acthar	
			Pre-Acthar	Post-Acthar % change (mg/dL)	Post-Acthar % change (mg/d)	
			Post-Acthar (g/dL)			
1	Prednisone, cyclosporine, cyclophosphamide	ACEI, cyclosporine	1.7	1.7	6700	Partial remission
			2.7	1.6	3300	
				−5.9	−50.7	
2	Prednisone, cyclosporine, MMF, tacrolimus, rituximab	Tacrolimus, MMF	3.0	0.9	5800	Partial remission
			3.4	0.8	2016	
				−11.1	−65.2	
3	Prednisone	None	3.2	2.2	5000	Clinical response
			3.8	2.2	3422	
				0	−31.6	
4	None	ARB	3.9	4.8	7900	Partial remission
			4.0	6.7	2300	
				39.6	−70.9	
5	None	ARB	3.2	3.2	3840	Early termination
			NA	NA	NA	
				NA	NA	
6	Prednisone, cyclosporine	ARB, ACEI cyclosporine	2.8	3.0	7500	Partial remission
			3.9	2.5	1768	
				−16.7	−76.4	
7	Prednisone, cyclosporine	ARB, ACEI cyclosporine	3.1	1.1	5280	Clinical response
			3.6	1.3	3560	
				18.2	−32.6	
8	None	ACEI	NA	1.2	4000	Partial remission
			NA	1.1	765	
				−8.3	−80.9	
9	Prednisolone, methotrexate	ACEI, prednisolone	3.1	1.6	9306	Partial remission
			3.3	2.0	2773	
				25.0	−70.2	
10	MMF	ACEI, MMF	NA	4.4	2830	Clinical response
			4.0	5.0	1629	
				13.6	−42.4	
11	Prednisone	ARB	3.5	2.4	3500	Partial remission
			3.7	3.1	750	
				29.2	−78.6	
12	Prednisone	ACEI	3.8	1.5	5700	Early termination (Partial remission)
			4.0	1.2	1500^a^	
				−20.0	−73.7	
13	Prednisone, cyclosporine	None	3.2	3.1	3250	Clinical response
			3.5	4.1	2073	
				32.3	−36.2	
14	Prednisone, cyclosporine	ACEI	3.7	2.5	2500	Partial remission
			NA	3.0	1246	
				20.0	−50.2	
15	Prednisone, cyclosporine, MMF	ACEI	3.6	1.4	4070	No response
			3.3	1.5	3930	
				7.1	−3.4	

*Abbreviations*: *ACEI* angiotensin-converting-enzyme inhibitor, *ARB* angiotensin II receptor blockers, *CT* cytotoxic therapy, *FSGS* idiopathic focal segmental glomerulosclerosis, *IST* immunosuppressive therapy, *MMF* mycophenolate mofetil, *NA* not available

^a^Post-Acthar gel assessment occurred following 4 months of treatment

**Table 3:** Proteinuria reduction and treatment response in patients with iMN treated with Acthar gel

Patient	Previous IST/CT	Concurrent medications	Serum albumin	SCr	Proteinuria	Treatment response
				Pre-Acthar	Pre-Acthar	
			Pre-Acthar	Post-Acthar % change (mg/dL)	Post-Acthar % change (mg/d)	
			Post-Acthar (g/dL)			
1	Prednisone, cyclophosphamide, rituximab	ARB, ACEI	2.7	1.3	13,600	Clinical response
			3.2	1.3	6600	
				0	−51.5	
2	Prednisone, IVMP	Prednisone	1.4	1.2	6354	Partial remission
			3.3	1.1	1000	
				−8.3	−84.3	
3	None	None	1.5	2.5	15,400	Partial remission
			3.1	1.9	2376	
				−24.0	−84.6	
4	Prednisone, cyclosporine	ACE	1.6	1.9	10,000	Clinical response
			2.1	1.9	4000	
				0	−60.0	
5	Prednisone	ARB, ACEI	3.8	1.9	4000	No response
			3.7	2.0	3475	
				5.3	−13.1	
6	Tacrolimus	ACEI	3.3	1.0	5500	Complete remission
			3.4	0.9	349	
				−10.0	−93.7	
7	Prednisone, tacrolimus, chlorambucil	ARB	3.9	0.9	2400	Complete remission
		Tacrolimus	4.0	1.0	163	
				−11.1	−93.2	
8	Prednisone, cyclosporine	ACEI	3.2	1.3	3070	Early termination
			NA	NA	NA	
				NA	NA	
9	Prednisone, cyclosporine	ACEI	3.8	2.1	1930	Partial remission
			3.7	2.2	728	
				4.8	−62.3	
10	Prednisone, tacrolimus, cyclophosphamide	ARB	3.5	1.3	5210	Partial remission
			3.7	1.6	1780	
				23.1	−65.8	
11	Prednisone, IVMP, cyclophosphamide	ACEI	NA	3.3	5132	No response
			NA	3.8	6600	
				15.2	28.6	

*Abbreviations*: *ACEI* angiotensin-converting-enzyme inhibitor, *ARB* angiotensin II receptor blockers, *CT* cytotoxic therapy, *iMN* idiopathic membranous nephropathy, *IST* immunosuppressive therapy, *IVMP* intravenous methylprednisolone, *NA* not available

**Table 4:** Proteinuria reduction and treatment response in patients with IgA nephropathy/diabetic nephropathy treated with Acthar gel

Patient	Previous IST/CT	Concurrent medications	Serum albumin	SCr	Proteinuria	Treatment response
			Pre-Acthar	Pre-Acthar	Pre-Acthar	
			Post-Acthar (g/dL)	Post-Acthar % change (mg/dL)	Post-Acthar % change (mg/d)	
IgAN						
1	None	ARB, ACEI	3.9	2.8	4000	Early termination
			NA	NA	NA	
				NA	NA	
2	None	ARB, ACEI	3.9	1.4	2674	Clinical response
			NA	1.5	1700	
				7.1	−36.4	
3	None	ARB	4.0	1.3	2439	No response
			3.6	1.3	2360	
				0	−3.2	
4	None	ACEI	3.0	1.0	10000	Early termination (Partial remission)
			4.0	1.0	800^a^	
				0	−92.0	
5	Prednisone, cyclophosphamide, azathioprine	ARB	NA	1.3	2230	Partial remission
			4.2	1.3	815	
				0	−63.5	
DN						
1	None	ACEI	2.0	1.9	25000	No response
			2.0	4.9	23000	
				157.9	−8.0	
2	None	ARB, ACEI	3.3	3.4	14000	Early termination
			3.3	4.4	11600^a^	
				29.4	−17.1	
3	None	ACEI	3.1	4.8	17570	No response
			2.8	5.7	18886	
				18.8	7.5	
4	None	ACEI	3.4	2.5	11000	Clinical response
			3.5	2.5	6895	
				0	−37.3	

*Abbreviations*: *ACEI* angiotensin-converting-enzyme inhibitor, *ARB* angiotensin II receptor blockers, *CT* cytotoxic therapy, *DN* diabetic nephropathy, *IgAN* IgA nephropathy, *IST* immunosuppressive therapy, *MMF* mycophenolate mofetil, *NA* not available

^a^Post-Acthar gel assessment occurred following 3 months of treatment

**Table 5:** Proteinuria reduction and treatment response in patients treated with Acthar gel, by etiologic diagnosis

Patient	Previous IST/CT	Concurrent medications	Serum albumin	SCr	Proteinuria	Treatment response
			Pre-Acthar	Pre-Acthar	Pre-Acthar	
			Post-Acthar (g/dL)	Post-Acthar % change (mg/dL)	Post-Acthar % change (mg/d)	
MLN						
1	Prednisone, cyclophosphamide	ACEI, prednisone	1.8	1.0	8000	Partial remission
			3.3	0.8	1089	
				−20.0	−86.4	
2	MMF	ACEI	1.7	1.0	19890	Partial remission
			2.4	1.1	2454	
				10.0	−87.7	
MCD						
1	Prednisone	ACEI	3.7	0.9	2000	Complete remission
			4.7	1.2	241	
				33.0	−88.0	
2	Prednisone, cyclosporine	ACEI	2.1	1.0	15000	Complete remission
			2.3	0.7	89	
				−30.0	−99.4	
FGN	Prednisone, MMF, rituximab	MMF	1.4	5.6	13000	No response
			3.4	9.0	10000	
				60.7	−23.1	
MPGN	None	ACEI	1.5	0.7	10000	Partial remission
			3.3	0.8	2141	
				14.3	−78.6	
OTHER^a^						
1 UNS	Prednisone	None	3.5	1.6	3000	Clinical response
			4.6	2.2	1600	
				37.5	−46.7	
2 UNS	Prednisone	ACEI	3.5	1.7	4500	Partial remission
			4.0	1.9	2000	
				11.8	−55.6	
3 UNS	None	None	3.1	1.3	5500	Early termination
			3.3	1.3	NA	
				0	NA	

*Abbreviations*: *ACEI* angiotensin-converting-enzyme inhibitor, *ARB* angiotensin II receptor blockers, *FGN* fibrillary glomerulonephritis, *MCD* minimal change disease, *MLN* membranous lupus nephritis (class V), *MPGN* membranoproliferative glomerulonephritis, *NA* not available, *UNS* unbiopsied nephrotic syndrome

^a^“Other” includes 3 patients with unbiopsied NS

### Total group treatment response

There was significant proteinuria reduction from baseline to post-Acthar gel treatment (*n* = 40; mean reduction 3984.8 ± 4069.1 mg/d, *P* < 0.0001). Total cholesterol showed significant decline from baseline to post-Acthar gel therapy (*n* = 21; mean reduction 38.3 ± 58.8 mg/dL, *P* = 0.007). Mean serum albumin at baseline indicated hypoalbuminemia (*n* = 40, 3.0 ± 0.8 g/dL; range 1.4–4.0 g/dL), and significant improvement was shown post-Acthar gel therapy (*n* = 35; mean improvement 0.53 ± 0.6 g/dL, *P* < 0.0001).

Acthar gel treatment was completed by 37 patients, and 7 (15.9 %) patients had early termination of treatment due to AEs. Among the 37 treatment completers, 81.1 % (30/37) showed ≥30 % proteinuria reduction, and 62.2 % (23/37) showed ≥50 % proteinuria reduction. Proteinuria remission was shown by 56.8 % (21/37) of patients, either complete (*n* = 4, 10.8 %) or partial (*n* = 17, 45.9 %) remission. Inclusion of clinical response patients (*n* = 9) resulted in 81.1 % (30/37) of patients showing substantial proteinuria reduction. Of these patients, 80 % (24/30) had failed ≥1 and 53.3 % (16/30) had failed ≥2 prior immunosuppressive or cytotoxic therapies.

Among the 44 patients, 26 (59.1 %) showed SCr >1.3 mg/dL at baseline. There was a greater mean percent proteinuria reduction in patients with SCr ≤1.3 mg/dL (*n* = 14; 72.2 ± 26.9 % reduction) compared with patients showing SCr >1.3 mg/dL (*n* = 22; 41.0 ± 29.7 % reduction, *P* = 0.0031). Similarly, among patients showing complete or partial remission or clinical response, greater mean percent proteinuria reduction occurred in patients without renal function impairment (*n* = 13; 77.5 ± 19.0 % reduction) compared with SCr >1.3 mg/dL (*n* = 16; 55.6 ± 16.9 % reduction, *P* = 0.0029).

### Treatment response by NS etiology

**Fig. 1:**
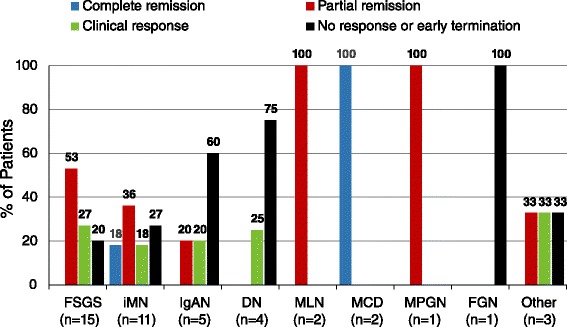
Treatment response in patients with NS treated with Acthar gel, by etiologic diagnosis. “Other” includes 3 patients with unbiopsied NS. Abbreviations: DN, diabetic nephropathy; FGN, fibrillary glomerulonephritis; FSGS, idiopathic focal segmental glomerulosclerosis; IgAN, IgA nephropathy; iMN, idiopathic membranous nephropathy; MCD, minimal change disease; MLN, membranous lupus nephritis (class V); MPGN, membranoproliferative glomerulonephritis

The percentage of patients showing complete remission, partial remission, or clinical response to Acthar gel treatment varied across NS etiologies (Fig. 1). The highest proteinuria responses were seen in patients with MCD (*n* = 2/2 complete remission), MLN (*n* = 2/2 partial remission), MPGN (*n* = 1/1 partial remission), FSGS (*n* = 12/15 [80.0 %] partial remission or clinical response), and iMN (*n* = 8/11 [72.7 %] complete or partial remission or clinical response). Lower proteinuria responses were seen in patients with IgAN (*n* = 2/5 [40.0 %] partial remission or clinical response) and DN (*n* = 1/4 [25 %] clinical response). The single patient with FGN showed no response. Within the “Other” category of unbiopsied NS patients, 1 patient showed partial remission and 1 patient showed clinical response.

#### FSGS

Following Acthar gel, 86.7 % (13/15) of patients showed ≥30 % proteinuria reduction and 60 % (9/15) showed ≥50 % proteinuria reduction (Table 2). There was significant proteinuria reduction from baseline to post-Acthar gel treatment (*n* = 14; mean reduction 3021.7 ± 1970.6 mg/d, *P* < 0.0001). No patient showed complete remission; however, 9 (60 %) showed partial remission, and 4 (26.7 %) showed clinical responses ranging from 31.6–42.4 % proteinuria reduction. One patient showed no response, and 2 patients had early termination. The early termination for 1 patient was due to increased swelling; no reason was provided for the second patient, who achieved partial remission prior to termination of treatment. Renal insufficiency at baseline due to SCr >1.3 mg/dL was shown in 12/15 (80 %) patients. Among the Acthar gel treatment responders, worsening SCr >25 % was shown in 2 patients with partial remission and 1 patient with clinical response and all 3 patients had SCr >1.3 at baseline (Table 2). Total cholesterol significantly declined from baseline (*n* = 8; mean reduction 32.5 ± 28.9 mg/dL, *P* < 0.02), and serum albumin significantly increased (*n* = 11; mean increase 0.39 ± 0.4 g/dL, *P* = 0.009). Hypoalbuminemia was shown by 8/15 patients (53.3 %) at baseline (range 1.7–3.2 g/dL), and improved in 7 of these patients by post-treatment (range 2.7–3.9 g/dL).

#### iMN

Proteinuria reduction ≥50 % occurred in 72.7 % (8/11) of patients with iMN (Table 3). There was significant proteinuria reduction from baseline to post-Acthar gel treatment (*n* = 10; mean reduction 4245.5 ± 4085.5 mg/d, *P* = 0.009). Two patients (18.2 %) showed complete remission, 4 (36.4 %) showed partial remission, and 2 (18.2 %) showed clinical responses ranging from 51.5–60.0 % proteinuria reduction. Two patients showed no response (1 patient was diagnosed with iMN and FSGS), and 1 patient had early termination due to an AE involving fatigue. SCr >1.3 mg/dL was found in 5/11 patients (45.5 %) at baseline. The mean reduction in total cholesterol (*n* = 5; 9.4 ± 10.2 mg/dL) and increase in serum albumin (*n* = 9; 0.52 ± 0.7 g/dL) were not significant (*P* > 0.06). Hypoalbuminemia was shown by 6/11 patients (54.5 %) at baseline (range 1.4–3.3 g/dL), and 5 of these patients showed improvement post-treatment (range 2.1–3.4 g/dL).

#### IgAN

Proteinuria reduction ≥30 % occurred in 60 % (3/5) of patients and 40 % (2/5) showed ≥50 % proteinuria reduction (Table 4). The mean proteinuria reduction (*n* = 4) was 2917.0 ± 4225.4 mg/d. Two patients showed partial remission and 1 patient showed clinical response (36.4 % proteinuria reduction). One patient showed no response and 2 patients had early termination. Early termination for 1 patient was due to AEs of weight gain and hypertension, and for 1 patient was stated as a patient decision. This latter patient showed partial remission, with 92 % proteinuria reduction before treatment termination. SCr >1.3 mg/dL occurred in 2/5 patients (40 %) at baseline. Total cholesterol and serum albumin were available for 2 patients. Total cholesterol decreased from 250 mg/dL to 230 mg/dL and serum albumin increased from 3.0 g/dL to 4.0 g/dL in 1 patient whereas the second patient had increased total cholesterol from 161 mg/dL to 242 mg/dL and decreased serum albumin from 4.0 g/dL to 3.6 g/dL. One patient showed hypoalbuminemia at baseline (3.0 g/dL) and improved post-treatment (4.0 g/dL).

#### DN

Among 4 patients, 1 (25 %) showed ≥30 % proteinuria reduction (37.3 %, Table 4). The mean proteinuria reduction (*n* = 4) was 1797.3 ± 2267.3 mg/d. Two patients had no response to treatment, including 1 patient with DN and FSGS who progressed to renal replacement therapy. One patient had early termination due to AEs involving weight gain and hypertension. All 4 patients showed SCr >1.3 mg/dL. There was a mean reduction in total cholesterol (*n* = 2; 104.5 ± 13.4 mg/dL) and in serum albumin (*n* = 4; 0.05 ± 0.2 g/dL). Hypoalbuminemia was shown by all 4 patients at baseline (range 2.0–3.4 g/dL) and improved to 3.5 g/dL in 1 patient.

#### MLN

Both patients with MLN showed ≥50 % proteinuria reduction (86.4 % and 87.7 %), and showed partial remission (Table 5). The mean proteinuria reduction (*n* = 2) was 12173.5 ± 7442.3 mg/d. The patients did not experience renal insufficiency, but they did show hypoalbuminemia at baseline (1.7 and 1.8 g/dL), which improved post-treatment (3.3 and 2.4 g/dL). There was a mean increase in serum albumin (*n* = 2; 1.10 ± 0.6 g/dL). Change in total cholesterol was not available for either patient.

#### MCD

Both patients with MCD showed ≥50 % proteinuria reduction (88 % and 99.4 %), and showed complete remission (Table 5). The mean proteinuria reduction (*n* = 2) was 8335.0 ± 9299.9 mg/d. Renal insufficiency was not present but 1 patient showed a worsening of SCr from 0.9 at baseline to 1.2 mg/dL (33 %). One patient showed hypoalbuminemia at baseline (2.1 g/dL) with minimal improvement post-treatment (2.3 g/dL). There was mean total cholesterol reduction (*n* = 2; 81.5 ± 2.1 mg/dL) and mean serum albumin increase (*n* = 2; 0.60 ± 0.6 g/dL).

#### FGN

The patient with FGN showed no response to treatment (Table 5). The patient had advanced disease that required renal replacement therapy, and dialysis was initiated before starting Acthar gel therapy.

#### MPGN

The patient with MPGN showed partial remission, with 78.6 % proteinuria reduction (Table 5). This patient did not show renal insufficiency but did show hypoalbuminemia at baseline (1.5 g/dL) that improved post-treatment (3.3 g/dL). Total cholesterol was reduced from 360 mg/dL to 180.0 mg/dL.

#### Unbiopsied NS

Among the 3 patients with unbiopsied NS, 1 patient showed partial remission (55.6 % proteinuria reduction), and 1 patient showed clinical response (46.7 % proteinuria reduction) (Table 5). The third patient had early termination of treatment due to an AE involving seizures. The patients with partial remission and clinical response showed renal insufficiency at baseline and the patient with a clinical response showed worsening of SCr >25 %. Hypoalbuminemia was present in the patient with early termination. The patient with partial remission had a total cholesterol reduction of 100 mg/dL.

### Safety and tolerability

AEs during Acthar gel treatment were reported by 29.5 % (13/44) of patients and included increased swelling, weight gain, hypertension, hyperglycemia, fatigue, dizziness, hypokalemia, upper respiratory infection, seizures, and decreased bone mineralization (Table 6). Early termination due to treatment-related AEs occurred in 15.9 % (7/44) of patients. Among these 7 patients, 1 patient with IgAN terminated treatment early by “patient decision” and 1 patient with FSGS terminated treatment early without a specific reason provided. Both patients showed partial remission before early termination (Table 2, Table 4).

**Table 6:** Adverse events and early termination of Acthar gel treatment in patients with NS

Nephrotic syndrome etiology	Patients reporting treatment-related AEs, *n* (%)	Treatment-related AEs	Early termination due to AEs^a^, *n* (%)
FSGS (*n* = 15)	3 (20 %)	Increased swelling (*n* = 1) Hyperglycemia (*n* = 2) Hypertension (*n* = 1) Weight gain (*n* = 1) Upper respiratory infections (*n* = 1)	1 (6.7 %) Edema 1 (6.7 %) Reason not given
iMN (*n* = 11)	4 (36.4 %)	Fatigue (*n* = 1) Dizziness (*n* = 1) Weight gain (*n* = 2) Hypokalemia (*n* = 1)	1 (9 %) Fatigue
IgAN (*n* = 5)	1 (20 %)	Weight gain (*n* = 1) Hypertension (*n* = 1)	1 (20 %) Weight gain, hypertension 1 (20 %) Patient decision
DN (*n* = 4)	3 (75 %)	Weight gain (*n* = 2) Hypertension (*n* = 1) Hyperglycemia (*n* = 1) Decreased bone mineralization (*n* = 1)	1 (25 %) Weight gain, hypertension
MLN (*n* = 2)	0		0
MCD (*n* = 2)	0		0
FGN (*n* = 1)	0		0
MPGN (*n* = 1)	0		0
Other^b^ (*n* = 3)	2 (66.7 %)	Seizures (*n* = 1) Hyperglycemia (*n* = 1) Weight gain (*n* = 1) Hypertension (*n* = 1)	1 (33.3 %) Seizures

*Abbreviations*: *AEs* adverse events, *DN* diabetic nephropathy, *FGN* fibrillary glomerulonephritis, *FSGS* idiopathic focal segmental glomerulosclerosis, *IgAN* IgA nephropathy, *iMN* idiopathic membranous nephropathy, *MCD* minimal change disease, *MLN* membranous lupus nephritis (SLE class V), *MPGN* membranoproliferative glomerulonephritis, *UNS* unbiopsied nephrotic syndrome

^a^Patients without a specific reason given for early termination of treatment were included in the count of early termination due to AEs

^b^“Other” includes 3 patients with unbiopsied NS

## Discussion

This retrospective case series is the largest published to date to examine the efficacy and safety of Acthar gel in the treatment of patients with NS of varying etiologies, 68.2 % of whom had received prior NS treatment with immunosuppressive or cytotoxic therapies, who were receiving clinic-based prescription treatment. A significant proteinuria reduction was shown, and approximately 80 % of patients who completed Acthar gel treatment showed a substantial proteinuria reduction of ≥30 %, including patients who met criteria for complete remission, partial remission, or clinical response. Most patients tolerated Acthar gel therapy well. The AEs were consistent with prior studies of Acthar gel in patients with NS, in which AEs were typically steroid-like, with most being mild to moderate in severity and transient [14–17].

The relative rarity of NS etiologies has contributed to the scarcity of large-scale, prospective, randomized, controlled trials on which to base treatment recommendations [1]. As a result, the majority (67 %) of the recommendations provided by the comprehensive Kidney Disease: Improving Global Outcomes (KDIGO) 2012 Clinical Practice Guideline for Glomerulonephritis were graded as a suggestion rather than recommendation and with an evidence quality rating of C or D, indicating low to very low quality of evidence [1]. Evaluation of additional treatment options is urgently needed for patients with FSGS, iMN, IgAN, MCD, and MLN who are not responsive to the first-line treatments—typically corticosteroids, cyclophosphamide, CNIs, and mycophenolate mofetil (MMF)—or who are unable to tolerate the first-line treatments. For example, approximately 25 % of patients with MCD have been shown to be steroid-resistant and approximately 30 % of initial steroid responders show frequent relapses, and approximately one-third of MLN patients have been shown not to respond to the current American College of Rheumatology (ACR)-recommended initial treatment with prednisone plus MMF [4, 7, 8]. While acknowledging retrospective case series study design limitations, the current large case series provides much-needed Acthar gel treatment response information in diverse patients, including patients with advanced disease and treatment-resistant NS. Half (53.3 %) of the patients who showed proteinuria response to Acthar gel had failed ≥2 prior immunosuppressive or cytotoxic therapies, and approximately half had impaired renal function. Among all patients and among patients who showed proteinuria reduction, patients with preserved renal function showed greater percent proteinuria reduction following Acthar gel treatment, indicating earlier treatment with Acthar gel may be especially beneficial.

Importantly, studies of patients with FSGS, iMN, and IgAN have indicated that partial remission and improved disease control are associated with better renal outcomes, even if patients relapse again [6, 21, 22]. Although complete remission is the ideal outcome, these studies suggest reduced proteinuria that does not meet complete remission criteria provides a meaningful treatment benefit compared with no improved disease control [6, 21, 22]. The optimal treatment duration for Acthar gel in patients with NS of varied etiology is not yet known. Our inclusion of the clinical response outcome identifying patients with substantial proteinuria reduction ≥30 % is consistent with the suggestion that longer-duration treatment beyond 6 months is indicated in iMN patients receiving CNIs who show proteinuria reduction of 30–50 %, with the goal of achieving partial or complete remission with longer-duration therapy [2]. Additionally, within the clinic, our experience with patients who show proteinuria reduction ≥30 % is clinically meaningful improvement in the patient’s report of feeling better. Longer-term treatment follow-up of these patients is needed to determine whether the proteinuria reduction is maintained, improves to remission or deteriorates to relapse.

In the two largest NS etiology patient groups, FSGS and iMN, the majority of patients showed proteinuria reduction ≥50 % following Acthar gel treatment, and the proteinuria reduction was significant. More than half of patients with FSGS showed partial remission and another quarter showed clinical response. Partial remission is a meaningful improvement for these patients, as significant improvement in kidney survival has been associated with partial remission of FSGS [14–16, 21]. Among patients with iMN, more than two-thirds of patients showed either complete or partial remission or clinical response to Acthar gel therapy. It has been stated that the recommended first-line therapy for patients with iMN, alkylating agents, should be restricted to patients who show a high risk of disease progression due to the toxicity associated with the agents [23]. Additionally, it has been shown that approximately 50 % of patients with iMN with persistent high-grade proteinuria will progress to end-stage renal disease (ESRD) [1]. The current case series findings, outcomes from previous studies examining Acthar gel in patients with iMN, and the finding that risk of progression is significantly reduced with at least partial remission suggest that Acthar gel may provide an important treatment option for patients with treatment-resistant iMN [14, 15, 19, 22].

The remaining NS etiology patient groups each had ≤5 patients, limiting conclusions about potential Acthar gel treatment efficacy. Proteinuria reduction to partial or complete remission was encouraging in patients with MCD or MLN and further study of Acthar gel therapy is warranted in these NS etiologies. Similarly, proteinuria response to Acthar gel therapy in 3 of the 5 patients with IgAN in our case series was consistent with prior single-case and small case series studies showing substantial proteinuria reduction [14, 15]. Compared with the 4 patients with DN in our case series, a stronger proteinuria response to Acthar gel therapy has been demonstrated in patients with DN using a treatment regimen of 16 U or 32 U daily for 6 months [17].

Potential mechanisms of action of Acthar gel include steroid-independent effects through the melanocortin system and steroid-related effects [13, 24, 25]. Acthar gel in an animal model of progressive renal tubulointerstitial injury showed suppression of tubulointerstitial inflammation, tubular atrophy, and fibrosis through anti-inflammatory effects mediated by melanocortin receptor 1 (MC1R) on tubular epithelial cells [26]. MC1Rs have been shown in podocytes, glomerular endothelial cells, and mesangial cells, and an MC1R agonist resulted in significantly reduced proteinuria in the passive Heymann nephritis animal model [24]. Thus, Acthar gel steroid-independent effects may occur through melanocortin receptors, and more specifically MC1R, which may provide an explanation for the efficacy of Acthar gel in treatment-resistant and steroid-resistant patients [24, 26]. Additionally, the cumulative dose of Acthar gel, through the dosing regimen and treatment duration, may be an important factor. Among iMN patients, those receiving a greater cumulative dose of Acthar gel (2800 U) showed greater proteinuria reduction compared with lower cumulative doses (880 U and 1760 U) [19].

The current report is the largest case series to date to examine Acthar gel treatment of patients in real-world, clinical nephrology practices with an all-inclusive patient population. Strengths include the large patient sample with diverse NS etiologies and the inclusion of a majority of patients with prior NS treatment and with impaired renal function. The real-world clinical practice of Acthar gel treatment in patients with NS helps to elucidate the AEs that may be expected. Limitations include the small patient numbers in several of the NS etiologies, the retrospective design without a control group, and the possibility that concurrent therapy or long-term effects of prior immunosuppressive or cytotoxic therapy may have contributed to the proteinuria response during Acthar gel therapy in some patients. Patients in the current study were primarily White, which may limit the applicability of study findings in more racially diverse populations. Initiation of anti-proteinuria treatment was based on the treating clinician’s judgment within the clinical management of their patient’s changing NS symptoms. As a result, excluding patients with IgAN, 8 patients began treatment at a non-nephrotic proteinuria level <3500 mg/d. Proteinuria was used as a surrogate endpoint and the possible long-term benefit of Acthar gel in preventing ESRD was not examined. Additionally, longer treatment duration and follow-up may be needed for optimal treatment responses. The relapse rate following successful treatment with Acthar gel and possible use of other therapies post-Acthar gel treatment cessation are not yet known.

## Conclusion

The current case series findings support a potential short-term benefit of Acthar gel therapy in patients with NS, particularly FSGS and iMN etiologies, and indicate Acthar gel treatment is well tolerated. Among the patients who completed ≥6 months of treatment, 80 % showed ≥30 % proteinuria reduction, and almost two-thirds showed ≥50 % proteinuria reduction. The majority of patients who showed proteinuria reduction had failed prior immunosuppressive and cytotoxic therapies, and approximately half showed impaired renal function prior to Acthar gel treatment. These findings indicate that Acthar gel may meet an important treatment need in patients with NS that is treatment-resistant in response to first-line therapies or who are unable to tolerate first-line therapies and in patients with advanced disease. Future research is needed to determine whether patients who show a proteinuria clinical response without remission benefit from longer-term treatment and show continued clinical improvement. Further research using prospective, controlled trials with longer-duration treatment and follow-up assessments to examine different Acthar gel regimens and cumulative dose effects in varied-etiology NS is warranted.

### Availability of supporting data

With the exception of total cholesterol, all raw data used in study summary analyses are provided in Tables 2, 3, 4, 5 and 6. Total cholesterol raw data are available on request.

## Abbreviations

ACEI: angiotensin-converting-enzyme inhibitor
ACR: American College of Rheumatology
ACTH: adrenocorticotropic hormone
AE: adverse event
ARB: angiotensin II receptor blocker
CNI: calcineurin inhibitor
DN: diabetic nephropathy
ESRD: end-stage renal disease
FGN: fibrillary glomerulonephritis
FSGS: idiopathic focal segmental glomerulosclerosis
IgAN: IgA nephropathy
iMN: idiopathic membranous nephropathy
KDIGO: kidney disease: improving global outcomes
MC1R: melanocortin receptor 1
MCD: minimal change disease
MLN: membranous lupus nephritis (SLE class V)
MMF: mycophenolate mofetil
MPGN: membranoproliferative glomerulonephritis
NS: nephrotic syndrome
SCr: serum creatinine
SLE: systemic lupus erythematosus
UPCR: urine protein:creatinine ratio
